# Geometric Morphometrics Reveals That Alfacalcidol, but Not Cholecalciferol, Preserves Renal Corpuscle Architecture in Rheumatoid Arthritis in Rats

**DOI:** 10.3390/ijms27010404

**Published:** 2025-12-30

**Authors:** Dina Kapić, Amela Dervišević, Samir Mehmedagić, Muhamed Katica, Asija Začiragić, Almir Fajkić, Aida Bešić, Nadža Kapo-Dolan, Gulali Aktas, Zurifa Ajanović

**Affiliations:** 1 Department of Histology and Embryology, Medical Faculty, University of Sarajevo, 71000 Sarajevo, Bosnia and Herzegovina; dina.kapic@mf.unsa.ba; 2 Department of Human Physiology, Medical Faculty, University of Sarajevo, 71000 Sarajevo, Bosnia and Herzegovina; amela.dervisevic@mf.unsa.ba (A.D.); asija.zaciragic@mf.unsa.ba (A.Z.); 3 Clinic for Heart, Blood Vessel and Rheumatic Diseases, Clinical Center University of Sarajevo, 71000 Sarajevo, Bosnia and Herzegovina; samir.mehmedagic@mf.unsa.ba; 4 Department of Clinical Sciences of Veterinary Medicine, Veterinary Faculty, University of Sarajevo, 71000 Sarajevo, Bosnia and Herzegovina; muhamed.katica@vfs.unsa.ba (M.K.); katicaaida1@gmail.com (A.B.); nadza.kapo@student.vfs.unsa.ba (N.K.-D.); 5 Department of Pathophysiology, Medical Faculty, University of Sarajevo, 71000 Sarajevo, Bosnia and Herzegovina; almir.fajkic@mf.unsa.ba; 6 Internal Medicine Department, Abant Izzet Baysal University Hospital, 14030 Bolu, Turkey; 7 Department of Anatomy, Medical Faculty, University of Sarajevo, 71000 Sarajevo, Bosnia and Herzegovina; zurifa.ajanovic@mf.unsa.ba

**Keywords:** rheumatoid arthritis, kidney, alfacalcidol, cholecalciferol, geometric morphometrics

## Abstract

Rheumatoid arthritis (RA) is a chronic autoimmune disease characterized by inflammation and destruction of cartilage, as well as by extra-articular manifestations. Rheumatoid nephropathy is a common complication of RA and its principal target is the renal corpuscle. Vitamin D and its analogs exert immunomodulatory actions throughout the body due to the widespread of their receptors. Our study aimed to compare the effects of cholecalciferol (vitamin D3) and alfacalcidol on renal corpuscle changes in pristane-induced RA model following a 28-day treatment, using geometric morphometrics. Forty female Wistar rats (190–210 g; 12–13 weeks old) were randomly assigned to four groups: the control (Cont) group (*n* = 10) received saline i.c., the PIA group (*n* = 10) was administered pristane i.c., PIA-ALF group (*n* = 10) was administered pristane i.c. and alfacalcidol orally, and the PIA-CH group (*n* = 10) was injected i.c. with pristane and received cholecalciferol orally. Pristane administration was used for RA induction. At the end of the experiment, the left kidneys were removed and processed by standard histological procedures for geometric morphometric analysis. Geometric morphometric analysis demonstrated that, compared with the control group, the architecture of the renal corpuscles was altered in the PIA (*p* < 0.0001) and PIA-CH (*p* = 0.0065) groups. In contrast, no statistically significant differences were observed in the PIA-ALF group (*p* = 0.3011). Geometric morphometric analysis demonstrated that alfacalcidol, but not cholecalciferol, exertedaprotective effect on the renal corpuscle architecture in pristane-induced rheumatoid arthritis in rats.

## 1. Introduction

Rheumatoid arthritis (RA) is a chronic inflammatory disease of unresolved etiology. It primarily affects joints, but can also cause extra-articular pathological changes. One of the principal extra-articular organs involved in RA is the kidney [1,2,3].

Rheumatoid nephropathy is considered one of the most serious complications of RA. Its severe forms can progress to chronic kidney disease [4,5]. It is estimated that about 25% of patients with RA experience chronic kidney disease [3]. The principal targets of RA renal damage are the renal corpuscles, and the morphological hallmarks of the rheumatoid glomerulopathy are mesangial expansion and hypercellularity, capillary loop irregularity and collapse, thickening of the glomerular basement membrane, glomerulosclerosis, and alteration in urinary space volume. This often results in scarring of the renal corpuscles that leads to their consequent shape alterations. As the rheumatoid glomerulopathy progresses, the number of functionally active nephrons decreases, which results in renal failure [4,5,6,7,8]. Despite the progress that has been made regarding the treatment of articular and extra-articular alterations of RA, effective treatments to prevent the development of RA nephropathy and consequent chronic kidney disease are still lacking [9].

Recent research suggests that vitamin D deficiency may increase the risk for RA development. It has been found that vitamin D has not only a function in the homeostasis of calcium and phosphate, but also exhibits immunomodulatory action on cells of both the innate and adaptive immune system. It regulates several genes involved in the functions of the immune system, influences the phagocytic and tumor cell cytotoxic activity of macrophages and reduces the production of different proinflammatory cytokines, such as tumor necrosis factor alpha (TNF-α) and interleukin 6 (IL-6) [10,11]. In clinical settings, vitamin D supplementation is used with increasing frequency in the treatment of RA, but the results of the available studies concerning its efficacy are contradictory [12]. There are different forms of vitamin D supplementation that are accessible for clinical use, both plain vitamin D and its analogs [13]. Cholecalciferol (vitamin D3) is a naturally occurring vitamin D form, obtained either by absorption from the digestive tube from dietary sources or synthesized in the skin upon UVB exposure. It is metabolized in the liver to calcidiol (25(OH)D3) and then in the kidneys to its biologically active form calcitriol (1,25(OH)2D3) [14]. Alfacalcidol is a synthetic vitamin D analog that is metabolized to calcitriol mainly in the liver. It bypasses metabolic processing by the 1α-hydroxylase in the kidney [15,16,17]. Compared to cholecalciferol, its onset of action is faster, and the risk of provoking hypercalcemia is lower [18].

Given the limited data on differences in the immunomodulatory effects of vitamin D and its analogs on renal morphology in RA, a direct comparison of cholecalciferol and alfacalcidol is warranted. Of particular interest is the evaluation of potential protective and tissue-preserving mechanisms before the occurrence of spontaneous resolution or unpredictable relapses at later stages of the disease.

We therefore hypothesized that alfacalcidol would provide superior protection against RA-induced glomerular injury compared to cholecalciferol. To test this, we employed geometric morphometrics, an image-based analytic method designed to quantify subtle, spatially complex changes in renal corpuscle architecture with high precision [19,20,21].

## 2. Results

### Principal Component (PC) Analysis of Renal Corpuscles

Table 1 shows the analysis of the principal components, as well as the degree of variability; the first seven principal components describe 96% of the variability, while the first principal component describes 59% of the variability.

**Figure 1:**
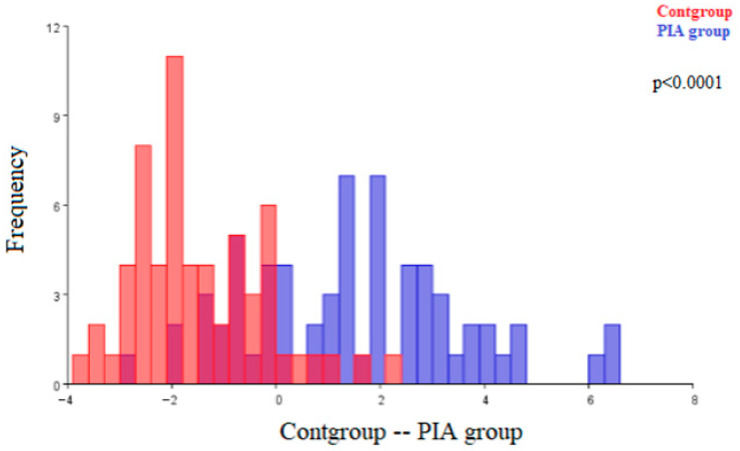
Discriminant function analysis of the morphological differences in renal corpuscles of the control (Cont) group and the pristane-induced arthritis (PIA) group. (Cont group—red; PIA group—blue). The two colors represent the compared groups, with overlapping regions indicating shared morphological features.

Figure 1 shows the architecture differences between the renal corpuscles of the control (Cont) group and the renal corpuscles of the pristane-induced arthritis (PIA) group. The observed morphological differences were statistically significant (*p* < 0.0001).

**Figure 2:**
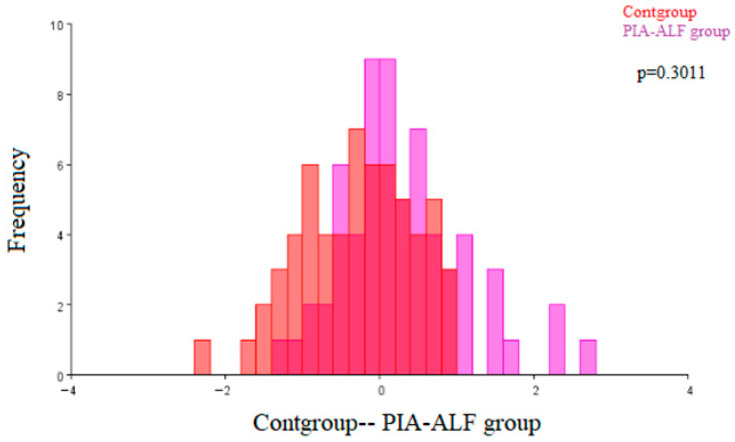
Discriminant function analysis of the morphological differences in the renal corpsucles of the control (Cont) group and the pristane-induced arthritis treated orally with alfacalcidiol (PIA-ALF) group. (Cont group—red; PIA-ALF group—purple).The two colors represent the compared groups, with overlapping regions indicating shared morphological features.

Figure 2 shows the results of the discriminant function analysis of the architecture differences in the renal corpuscles between the control (Cont) group of animals and the PIA-ALF group of animals. The observed morphological differences were not statistically significant (*p* = 0.3011).

**Figure 3:**
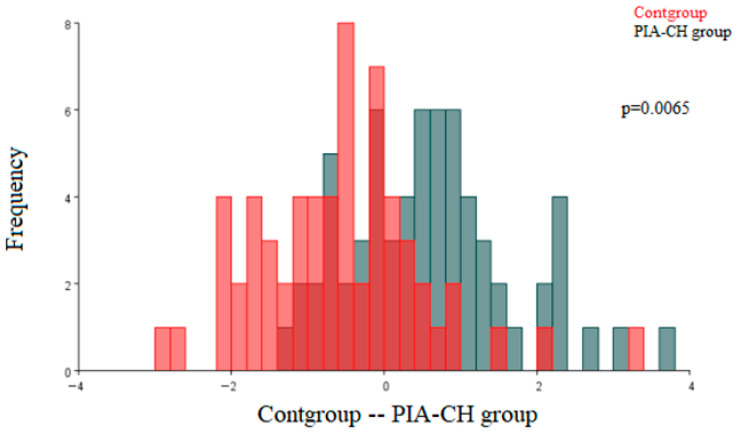
Discriminant function analysis of the architecture differences in the renal corpuscles of the control (Cont) group and the pristane-induced arthritis treated orally with cholecalciferol (PIA-CH) group. (Cont group—red; PIA-CH group—gray).The two colors represent the compared groups, with overlapping regions indicating shared morphological features.

Figure 3 shows the results of the discriminant function analysis of the architecture differences in the renal corpuscles between the control (Cont) group and the renal corpuscles of the PIA-CH group of animals. Statistically significant morphological differences were observed (*p* = 0.0065).

**Figure 4:**
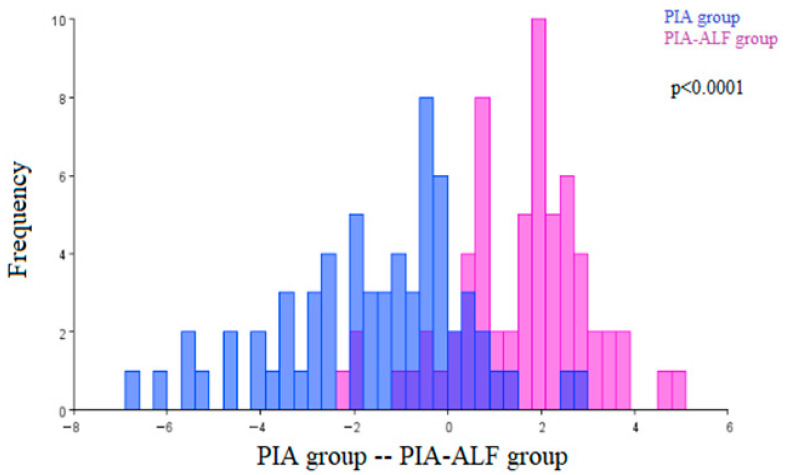
Discriminant function analysis of the architecture differences in the renal corpuscles of the pristane-induced arthritis (PIA) group and the pristane-induced arthritis treated orally with alfacalcidiol (PIA-ALF) group. (PIA—blue; PIA-ALF group—purple).The two colors represent the compared groups, with overlapping regions indicating shared morphological features.

In comparing the morphological characteristics of the renal corpuscles of the pristane-induced arthritis (PIA) group with the PIA-ALF group of animals, statistically significant architecture differences were observed (*p* < 0.0001) (Figure 4).

**Figure 5:**
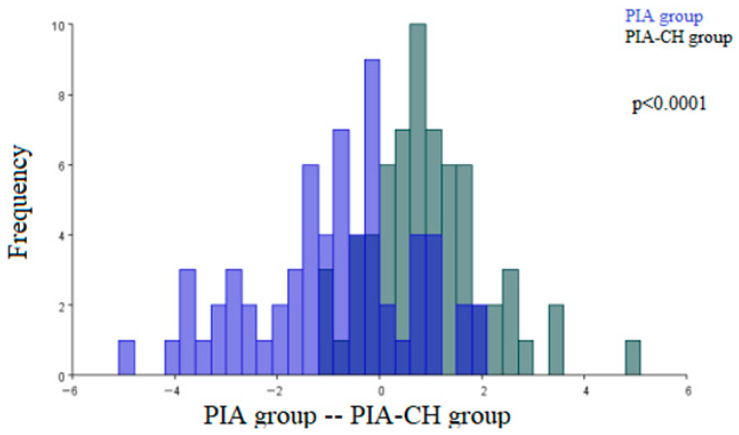
Discriminant function analysis of the architecture differences in the pristane-induced arthritis (PIA) group and the pristane-induced arthritis treated orally with cholecalciferol (PIA-CH) group. (PIA group—blue; PIA-CH group—gray).The two colors represent the compared groups, with overlapping regions indicating shared morphological features.

In comparing the morphological characteristics of the renal corpuscles of the PIA group with the renal corpuscles of the PIA-CH group, statistically significant architecture differences were observed (*p* < 0.0001) (Figure 5).

**Figure 6:**
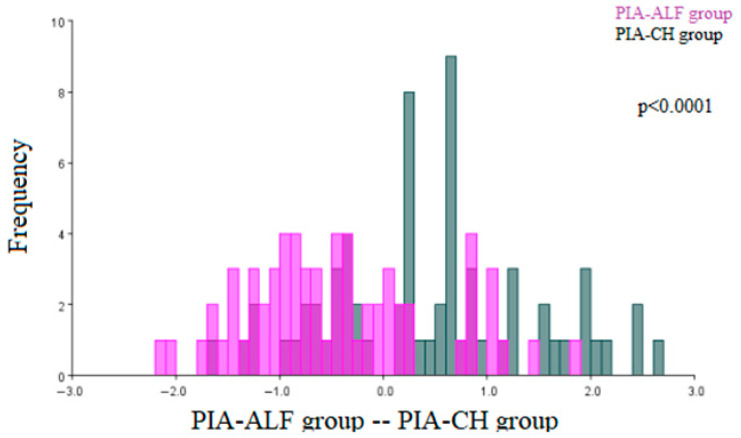
Discriminant function analysis of the architecture differences in the renal corpuscles of the pristane-induced arthritis treated orally with alfacalcidiol (PIA-ALF) group and the pristane-induced arthritis treated orally with cholecalciferol (PIA-CH) group. (PIA-ALF—purple; PIA-CH -gray).The two colors represent the compared groups, with overlapping regions indicating shared morphological features.

The comparison of the morphological characteristics of the renal corpuscles of the PIA-ALF group and PIA-CH group. Statistically significant architecture differences were observed (*p* < 0.0001), and the results of the discriminant function analysis are shown in Figure 6.

## 3. Discussion

To our knowledge, this is the first study to explore the potential protective effects of two different vitamin D chemical forms, alfacalcidol and cholecalciferol, on the alterations of renal corpuscles in RA. This study specifically addresses whether these two vitamin D analogs differ in their ability to prevent RA-induced glomerular injury, an area where clinical and experimental evidence remain inconclusive.

Beyond its well-known role in calcium-phosphate homeostasis, vitamin D exerts multiple physiological functions, including immunomodulatory effects. By binding to its receptor on immune cells, vitamin D changes gene expression and influences innate and adaptive immune system responses [11]. Deficiency of vitamin D has been linked to the pathogenesis of RA [22]. RA is a chronic autoimmune disorder characterized not only by joint inflammation and destruction but also by extra-articular manifestations, including renal alterations [2,3]. Kidney damage in RA has a progressive course, and chronic kidney disease develops in one-fourth of RA patients [23]. Higher RA activity has been associated with faster kidney function deterioration [24]. Because the level of proteinuria and the clinical manifestations do not always correspond precisely to the histopathological alterations, a biopsy assessment is often required in patients with unexplained renal function alteration to determine the diagnosis and initiate appropriate clinical management [25,26].

In our study, geometric morphometrics demonstrated clear architecture differences between the renal corpuscles of healthy control rats and untreated RA rats, confirming the efficacy of our animal RA model in inducing renal injury.

Biopsy studies in RA patients have reported that the renal corpuscle is the primary structure affected, exhibiting a range of histopathological patterns, most commonly mesangial proliferative glomerulonephritis, AA amyloidosis, and membranous nephropathy [25,26,27]. Key glomerular changes include mesangial expansion due to mesangial cell proliferation and excessive accumulation of mesangial matrix, glomerulosclerosis, and distortion of glomerular capillaries. Such alterations result in shape changes in the renal corpuscle [26], which were confirmed by our analysis.

Cytokines such as IL-6 and TNF-α play key roles in RA-related renal injury [28]. Rops et al. [29] showed that high levels of IL-6 are associated with mesangial proliferation and segmental podocyte foot process effacement, whereas IL-6 deficiency protects against glomerular damage in Cd37–/– mice. IL-6 can promote mesangial cell proliferation and interstitial fibrosis by enhancing the responsiveness of tubular epithelial cells to profibrotic mediators such as transforming growth factor-beta (TGF-β), and may contribute to kidney injury [30]. TNF-α is expressed in glomerular and tubular cells during inflammation [31], and elevated serum levels of TNF-α and soluble TNF receptors have been associated with the progression of kidney damage in different conditions, such as diabetic nephropathy, chronic kidney disease, aminoglycoside- and cisplatin-induced nephrotoxicity, and in ischemia–reperfusion kidney injury [32,33,34]. Furthermore, there is a positive correlation between high concentrations of TNF-α and the severity of kidney injury [35]. Our findings of altered glomerular morphology in untreated RA animals likely reflect these cytokine-driven pathways, linking chronic inflammation to progressive glomerulosclerosis.

Amyloidosis is characterized by deposition of fibrils, formed by aggregation of the acute phase serum amyloid A proteins, in renal tissue [36]. CRP can make deposits in the renal tubules and contribute to the early development of renal inflammation and fibrosis [37]. Genetic or pharmacological inhibition of TNF-α has been shown to mitigate renal injury in different conditions, which shows that the inflammatory pathways have an important role in kidney pathology [38]. Previous research has revealed that vitamin D supplementation decreases the TNF-α and IL-6 concentration in the blood [39,40]. It has also been demonstrated that vitamin D plays a role in the regulation of inflammation in allograft tissue in kidney transplant recipients, atherosclerosis, inflammatory bowel diseases, multiple sclerosis, diabetes and other conditions [41,42].

In our study, renal corpuscles of the alfacalcidol-treated RA animals did not significantly differ in architecture in comparison to those of the control animals, indicating that the treatment with alfacalcidol exerted a protective effect. In contrast to alfacalcidol, in RA animals treated with cholecalciferol, the renal corpuscles showed significant alterations in architecture in comparison to those of the healthy control animals, but exhibited no significant differences compared to the untreated RA animals. While cholecalciferol treatment led to a significant improvement compared with untreated PIA animals, the residual difference from controls suggests that the biological relevance of this effect is limited. In contrast, alfacalcidol fully prevented RA-induced corpuscular alterations, supporting its superior protective potential.

Previous studies have explored the protective effect of vitamin D in various conditions and the reported efficacy of cholecalciferol and alfacalcidol has been contradictory. Sonneveld et al. [43] demonstrated that 1,25-vitamin D3-deficient 25-hydroxy-vitamin-D3–1α-hydroxylase knockout mice and 1,25-vitamin D3-deficient rats develop podocyte injury and renal dysfunction. Changes in expression of podocin and nephrin, as well as podocyte process effacement were observed in 1,25-vitamin D3-deficient animals. Administration of 1,25-vitamin D3 or 1,25-vitamin D2 prevented or even reversed the podocyte alterations. Hamdy et al. [44] have explored the effect of early alfacalcidol administration on the development of renal bone disease in patients with mild to moderate chronic renal failure. They have demonstrated that alfacalcidol safely and effectively alters the natural course of the disease. Similarly, in the study of Shoji et al. [45] use of oral alfacalcidol was associated with reduced risk for cardiovascular death in end-stage renal disease patients, supporting our findings regarding the protective potential of alfacalcidol.

Only a few studies have compared the effects of alfacalcidol with other vitamin D chemical forms in different conditions. One such study has been performed by Matuszkiewicz-Rowińska et al. [46]. They have compared the effects of cholecalciferol (12.000 IU/week) and alfacalcidol (1.5 μg/week) in CKD patients. They concluded that cholecalciferol more effectively corrected 25(OH)D deficiency than alfacalcidol. This does not align with our findings, but this study measured only the correction of 25(OH)D levels, without assessing the downstream effects like the anti-inflammatory action. In contrast to that, the results of Scharla et al. [47] on RA-induced bone loss are in line with our findings. They demonstrated the beneficial action of alfacalcidol on renal changes in RA patients. They showed that alfacalcidol had a more potent effect on inflammation-induced bone loss than plain vitamin D.

The present study has some limitations to mention. First, pristane-induced arthritis in rats may not fully replicate the complexity and heterogeneity of human rheumatoid arthritis and its renal complications. Second, we used only female rats in the work so generalizability regarding sex-related biological differences is limited. Third, renal evaluations were performed only at the end of the experiment, making it impossible to track progression or temporal effects of treatments. Fourth, we tested only one dose and treatment duration in the work, thus conclusions on optimal dosing or therapeutic window was not possible.

## 4. Materials and Methods

### 4.1. Animals

Forty female Wistar rats (12–13 weeks old, weighing 190 to 220 g) were used in this study. They were housed in standard cages in a temperature-controlled room (23 ± 3 °C) with controlled humidity (50 ± 10%) under a 12 h dark/light cycle and maintained in an environment free of pathogenic microorganisms. Tap water and commercial rodent food were available ad libitum. Monitoring and supervision were performed daily by a team of competent and qualified personnel. The rats were randomly assigned to four groups:

1. Control group of healthy animals treated intracutaneously (i.c.) with saline solution (0.95% NaCl)—Cont group (*n* = 10),
2. Pristane-induced arthritis rats—PIA group (*n* = 10),
3. Pristane-induced arthritis rats treated orally with alfacalcidiol—PIA-ALF group (*n* = 10),
4. Pristane-induced arthritis rats treated orally with cholecalciferol—PIA-CH group (*n* = 10).

### 4.2. Procedure of Arthritis Induction

Rats were placed in a supine position, with their limbs restrained. The dorsal area at the base of the tail was sterilized using 70% ethanol. 150 µL of pristane (Acros Organics Pharmaceuticalaan 3, 2440 Geel, Belgium) was administered i.c. using a fine needle (27G) [48]. The control group underwent the same procedure and received 150 µL of saline (0.95% NaCl) at the same anatomical site.

Rheumatoid arthritis induction was confirmed using arthritis score, mobility score, ankle joint width, and joint hyperemia.

### 4.3. Application of Vitamin D

Cholecalciferol (Plivit D_3_ 4000 IU/mL–oral drops; manufacturer: Pliva–Croatia) was administered via oral gavage at a dose of 0.5 mL/day (500 IU/day) to the animals of the experimental group PIA-CH once daily for 28 consecutive days. The oral gavage was performed in the morning hours (8:00–10:00 a.m.). The administered solution (1000 IU/mL) was prepared by dissolving 5 mL of the cholecalciferol solution (Plivit D_3_ 4000 IU/mL) in 15 mL of saline. Alfacalcidol (Einsalpha^®^ 2 µg/mL–oral drops; manufacturer: CHEPLAPHARM Arzneimittel GmbH, Ziegelhof 24, 17489 Greifswald, Germany), was also administered via oral gavage at a dose of 0.2 µg/kg/day to the animals of the experimental PIA-ALF group once daily for a total of 28 days.The selected doses of cholecalciferol and alfacalcidol were based on previously published experimental protocols demonstrating immunomodulatory effects without inducing hypercalcemia [49,50], and approximate the upper physiological range for translational relevance to human RA studies.

### 4.4. Geometric Morphometrics

At the end of the experiment, left kidneys were collected, fixed in 10% buffered formalin, embedded in paraffin, sectioned at 5 µm thickness, and stained with hematoxylin-eosin for analysis under a light microscope. Representative images of the stained sections were taken and used as two-dimensional models for geometric morphometric analysis. These models were used to analyze the architecture differences in the renal corpuscles between the observed groups. Each model was marked with a numerical code containing information about the group, serial number of the animal and part of the kidney (upper, middle or lower pole) from which the sample came. The resulting images were then processed in the tpsUtil program and converted to tps format, after which they were imported into the tpsDig software (both programs were used in version 2.32, F. James Rohlf, Stony Brook, New York, United States of America 2016).

**Figure A1:**
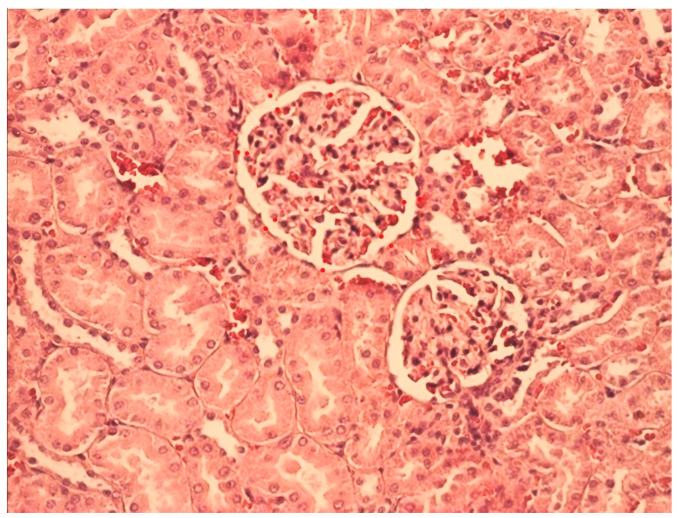
Specific points (landmarks) marked on the kidney glomerulus in the tpsDig program (H&E staining, magnification ×100).

This software was used to label certain specific points (landmarks) on the models. The order of entering the points was the same on all models, which ensured the consistency and validity of the geometric morphometric analysis. A total of 16 such points were recorded on each glomerulus-eight on the outer side of the glomerulus, and the other eight on the parietal layer of Bowman’s capsule (Figure A1, Appendix A).

### 4.5. Morphometric Data Analysis Procedures

In the MorphoJ program, morphological differences in renal corpuscles between individual groups of examined animals were analyzed on two-dimensional models of histologic slides.

**Figure A2:**
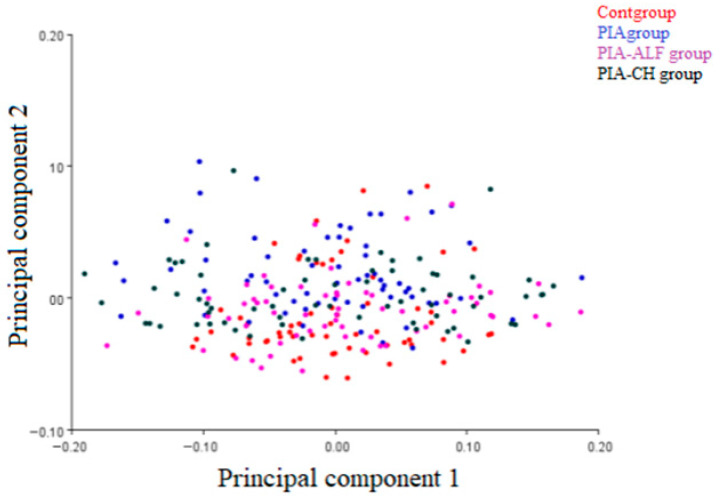
The position of the experimental animals according to the architecture of the renal corpuscles in the morphological space described by the first two main components (PC1 and PC2) arranged according to the corresponding groups.

After the generalized Procrustes analysis, analysis of the main components, introduction of the group as a classifier, a discriminant functional analysis was conducted that compared the morphological characteristics of the renal corpuscles between the examined groups of experimental animals and determined whether the established architecture differences were statistically significant. The position of the experimental animals, based on the architecture of the renal corpuscles, in the morphological space defined by the first two principal components (PC1 and PC2), distributed according to the corresponding groups, is shown in Figure A2, Appendix A.

### 4.6. Statistical Analysis

Data on the coordinates of all marked points were saved in tps files and further analyzed in the MorphoJ program (version 1.08.02, Klingenberg CP. London, UK, 2011). The MorphoJ program allowed the performance of standard tests of geometric morphometrics, which are used to identify the architectural differences in the examined structures. After importing the tps files into MorphoJ, analyses were performed based on the coordinates of the marked points. Tests of generalized Procrustes analysis, principal component analysis, discriminant functional analysis, and cross-validation were used. Discriminant analysis was used to compare morphological differences between the groups and determine the statistical significance of the observed differences. The results are depicted using graphs.

## 5. Conclusions

Geometric morphometric analysis demonstrated that pristane-induced arthritis was associated with significant alterations in renal corpuscle architecture. Treatment with alfacalcidol, but not cholecalciferol, preserved renal corpuscle architecture, showing no statistically significant difference compared with the control group. Further research should focus on clarifying the mechanisms underlying these protective effects and on determining optimal dosing strategies for clinical application.

## Figures and Tables

**Table 1:** Principal components (PC) analysis and degree of variability.

PCs	Eigenvalues	Variance %	Cumulative %
1.	0.00603319	58.848	58.848
2.	0.00097686	9.528	68.376
3.	0.00081066	7.907	76.283
4.	0.00063540	6.198	82.481
5.	0.00055292	5.393	87.874
6.	0.00050954	4.970	92.844
7.	0.00031132	3.037	95.881
8.	0.00010146	0.990	96.871
9.	0.00008040	0.784	97.655
10.	0.00007106	0.693	98.348
11.	0.00006986	0.681	99.029
12.	0.00003824	0.373	99.402
13.	0.00003204	0.313	99.715
14.	0.00002922	0.285	100.000

## Data Availability

The original contributions presented in this study are included in the article Material. Further inquiries can be directed to the corresponding author.

## Abbreviations

The following abbreviations are used in this manuscript:

RA	Rheumatoid arthritis
TNF-α	Tumor necrosis factor alpha
IL-6	Interleukin 6
25(OH)D3	Calcidiol
1,25(OH)2D3	Calcitriol
PC	Principal Component
BUN	Blood urea nitrogen
i.c.	Intracutaneously
Cont	Control
PIA	Pristane-induced arthritis
ALF	Alfacalcidiol
CH	Cholecalciferol
